# Unusual Presentation of Type 1 Idiopathic Macular Telangiectasia

**DOI:** 10.1155/2017/5395069

**Published:** 2017-01-19

**Authors:** Zaïnab Bentaleb Machkour, Philippe Denis, Laurent Kodjikian

**Affiliations:** ^1^Department of Ophthalmology, Croix-Rousse University Hospital, Hospices Civils de Lyon, University of Lyon I, 69004 Lyon, France; ^2^CNRS UMR 5510 Mateis, 69621 Villeurbanne, France

## Abstract

*Purpose*. To report unusual presentation of type 1A idiopathic macular telangiectasia (IMT).* Methods*. Two middle-aged women with bilateral IMT were examined.* Results*. Both patients presented with a gradual vision loss in both eyes. Fundus examination was unremarkable in one case and showed small macular telangiectasia in both eyes in the other case. Fluorescein angiography (FA) revealed early bilateral macular punctuated hyperfluorescence corresponding to the dilated capillaries in both cases. FA and fundus examination confirmed also the absence of vascular abnormalities in the middle or anterior fundus periphery in one case. Spectral-domain optical coherence tomography (SD-OCT) showed cystoid macular edema in both cases. No signs of retinal vein occlusions were detected in both cases and other differential diagnoses were excluded. Based on these findings, the patients were diagnosed with bilateral type 1A IMT according to Gass and Blodi classification and were treated with intravitreal antivascular endothelial growth factor (anti-VEGF) injections and focal laser photocoagulation. Twelve months later, SD-OCT revealed partial regression of the exudative signs and significant VA improvement.* Conclusion*. We described two patients with an unusual presentation of type 1A IMT with bilateral presentation, affecting two middle-aged women, with occult and without peripheral involvement in one case. The description of more cases of bilateral type 1 IMT should be helpful to more precisely define the pathophysiologic mechanism that could be different from a localized Coats' disease of the macula area.

## 1. Introduction

Idiopathic macular telangiectasia (IMT) was first described by Gass and Oyakawa in 1982 as juxtafoveal capillary abnormalities and dilatations [1]. In 1993, Gass and Blodi examined 140 cases over a 28-year period and established a classification of the entities with three groups, each one composed by subgroups [2]. Type 1 is characterized by unilateral macular telangiectasia with visible aneurysms and affects mainly young males. In this case, vision loss is due to macular edema formation. Type 1 includes two subtypes: type 1A IMT, the second most common type, characterized by visible and exudative telangiectasia and type 1B IMT, with visible, exudative, and focal telangiectasia limited to 2 clock hours or less in the juxtafoveal areas. Type 2 affects men and women and corresponds to bilateral acquired macular telangiectasia including two subtypes: type 2A IMT, affecting middle-age people, corresponds to occult and nonexudative telangiectasia and type 2B IMT corresponds to juvenile occult and familial telangiectasia. In this case the main complication includes choroidal neovascularization that may result in visual loss. Type 3 is very rare and includes occlusive bilateral macular telangiectasia associated with systemic or cerebral familial disease. More recently, Yannuzzi et al. proposed a more simplified classification of IMT and merged the type 1A and 1B IMT subgroups into group 1 aneurysmal telangiectasia and defined type 2A IMT as group 2 perifoveal telangiectasia [3]. Both classifications assess that type 1 IMT affects mainly young male and are defined as typically unilateral visible exudative telangiectasia corresponding to a localized form of Coats' disease in the macula area. We are reporting the case of two women presenting unusual type 1A IMT with bilateral involvement and suggesting the possibility of another pathophysiologic mechanism than a localized Coats' disease.

## 2. Case Reports

### 2.1. Case  1

**Figure 1:**
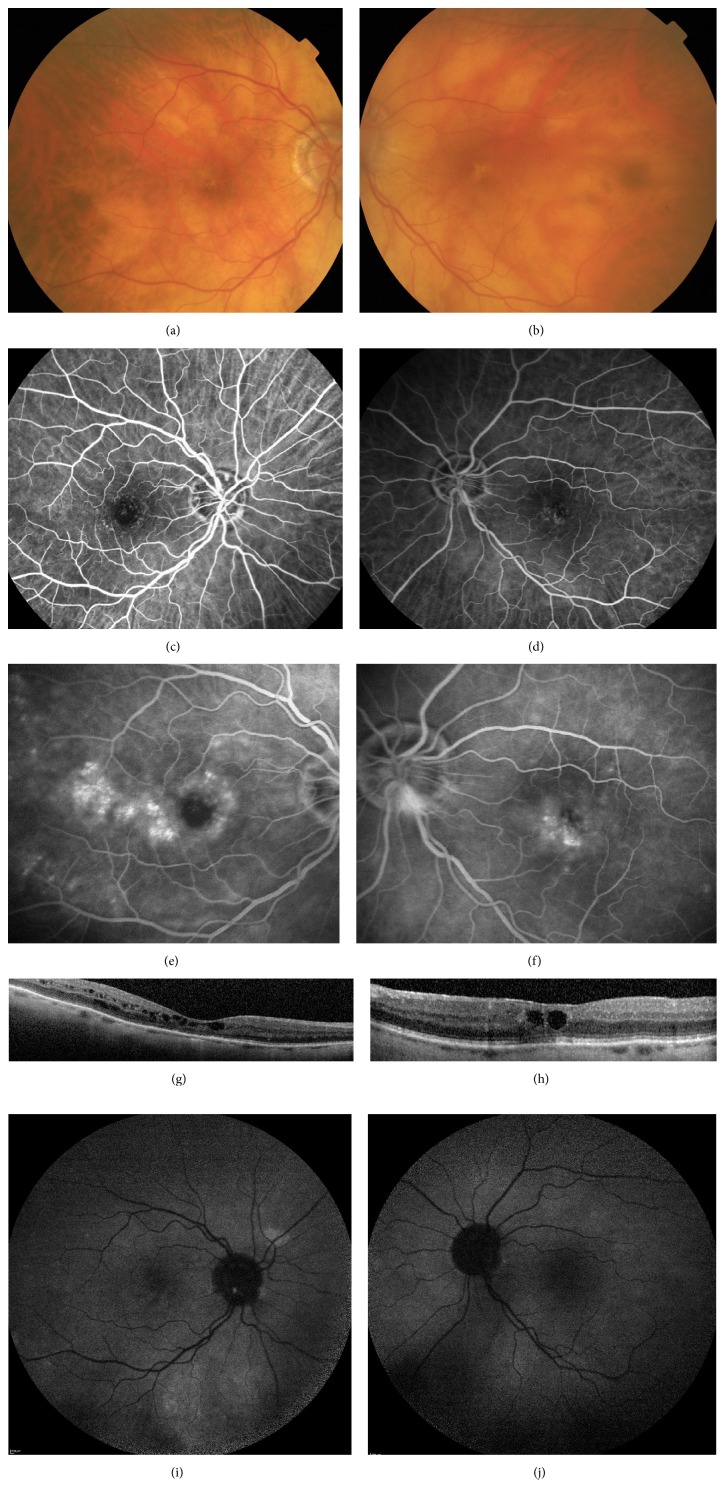
Patient 1. (a), (c), (e), and (g): right eye. (b), (d), (f), and (h): left eye. (a) and (b): fundus examination showing small pigment epithelium alterations in the foveal area without obvious vascular abnormalities. (c), (d), (e), and (f): fluorescein angiography (min and min) showing perifoveal telangiectasia with late intraretinal staining. (g) and (h): spectral-domain optical coherence tomography (B-scan) showing cystoid macular edema with a central macular thickness measured to 312 *μ*m on the right eye and to 345 *μ*m on the left eye. (i) and (j): autofluorescence images were unremarkable.

An 82-year-old woman presented at our clinic complaining of gradual vision loss in her both eyes since several weeks. Her past medical history included uncomplicated cataract surgery 18 years ago on both eyes. There was no medical history of diabetes or arterial hypertension. Her best-corrected visual acuity (BCVA) was 42 letters in the right eye (RE) and 61 letters in the left eye (LE). The anterior segment examination was unremarkable. Fundus examination revealed small pigment epithelium alterations in the foveal area without capillary abnormalities or dilatations on both eyes (Figures 1(a) and 1(b)). Early-phase fluorescein angiography (FA; Spectralis, HRA, Heidelberg Engineering, Germany) showed perifoveal telangiectasia with late intraretinal staining. FA confirmed also the absence of late staining of the optic disc and the absence of vein abnormalities excluding an Irvine-Gass syndrome or a branch retinal vein occlusion (Figures 1(c), 1(d), 1(e), and 1(f)). No vascular abnormalities in the middle or anterior fundus periphery were found on FA examination. SD-OCT (Spectralis Heidelberg Engineering, Germany) showed cystoid macular edema with a central macular thickness measured to 312 *μ*m RE and to 345 *μ*m LE (Figures 1(g) and 1(h)). Autofluorescence images were unremarkable on both eyes (Figures 1(i) and 1(j)).

Since there was no medical history of diabetes, arterial hypertension, or X-ray radiation and considering the absence of evidence of Irvine-Gass syndrome or retinal vein occlusion on FA examination we conclude the diagnosis of bilateral type 1 IMT. Moreover the absence of typical intraretinal pseudocysts on OCT rendered the diagnosis of type 2 IMT not compatible.

The patient was treated with anti-VEGF intravitreal injections in both eyes according to a PRN regimen associated with focal laser photocoagulation on telangiectasia located more than 1000 *μ*m to the fovea in the temporal area of her RE. This treatment allowed almost complete macular edema resorption with functional stability. Laser focal photocoagulation allowed increasing of the interval of injections during followup and the patient required less injections in the RE thereafter.

### 2.2. Case  2

**Figure 2:**
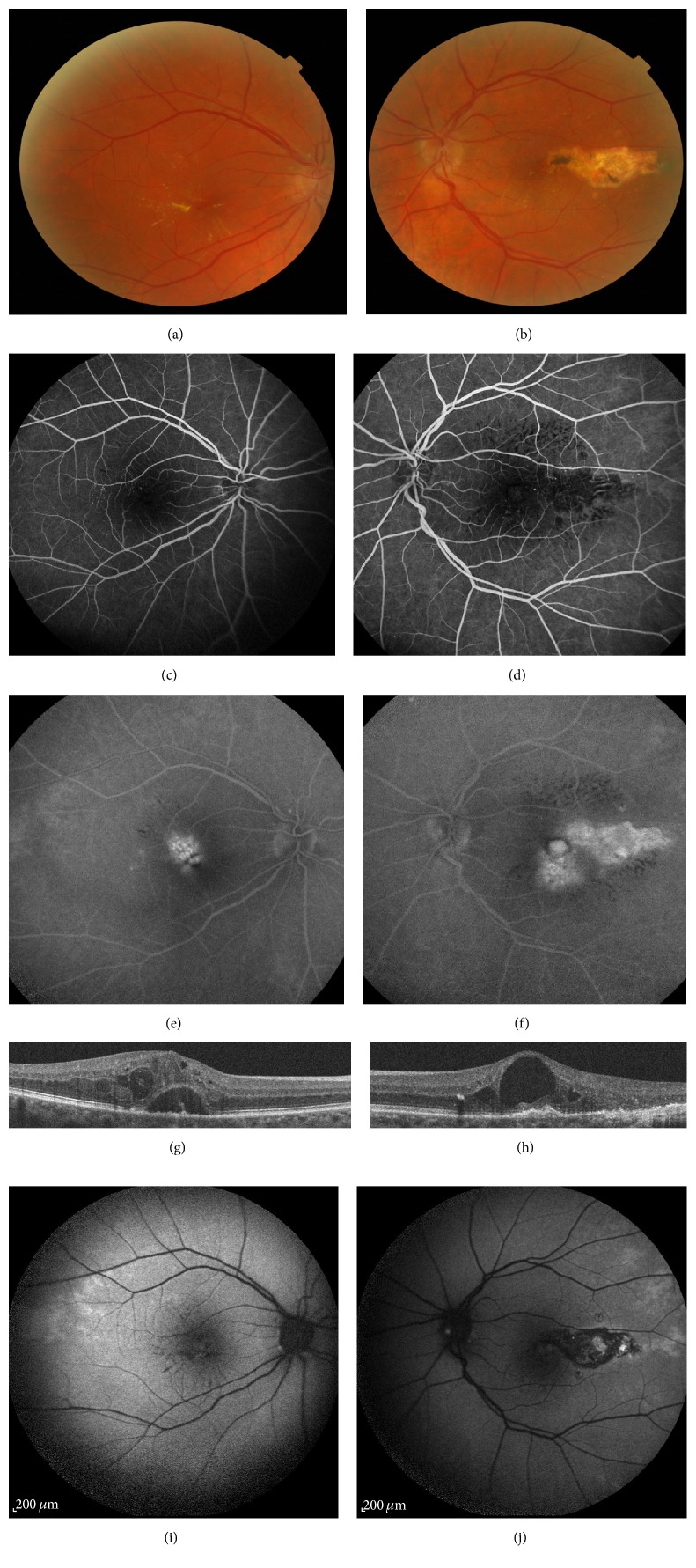
Patient 2. (a), (c), (e), and (g): right eye. (b), (d), (f), and (h): left eye. (a) and (b): fundus examination showing perifoveal exudates with small perifoveal capillary abnormalities on both eyes and a photocoagulation scar in the temporal area in the left eye. (c), (d), (e), and (f): fluorescein angiography (min and min) showing perifoveal telangiectasia mainly located in the superior and temporal area in the right eye and in the inferior and temporal area in the left eye with late intraretinal staining. (g) and (h): spectral-domain optical coherence tomography (B-scan) showing cystoid macular edema on both eyes with subretinal fluid in the right eye with a central macular thickness measured to 538 *μ*m on the right eye and to 334 *μ*m on the left eye. (i) and (j): autofluorescence images revealed in RE some hypoautofluorescent lesions in the temporal and inferior macular area, with a stellar distribution corresponding to the exudates. In the LE, the autofluorescence images revealed a large hypoautofluorescent lesion in the temporal area coexisting with smaller ones in the inferior area corresponding to photocoagulation scars.

A 60-year-old phakic woman presented at our clinic complaining of gradual vision loss in her both eyes since one year. She previously received 6 anti-VEGF and one dexamethasone intravitreal injections in her RE and 8 anti-VEGF and one dexamethasone intravitreal injections in her LE. She was also treated with focal laser photocoagulation in the macular temporal area on her LE during last one year. Her past medical history included also well-balanced arterial hypertension and no medical history of diabetes was found. Her best-corrected visual acuity (BCVA) was 74 letters in the right eye (RE) and 45 letters in the left eye (LE). The anterior segment examination was unremarkable. Fundus examination revealed perifoveal exudates with small perifoveal capillary abnormalities on both eyes (Figures 2(a) and 2(b)). Left eye examination showed also photocoagulation scar in the temporal area (Figure 2(b)). Some telangiectasia was found in anterior periphery on fundus examination. Early-phase FA (FA; Spectralis, HRA, Heidelberg Engineering, Germany) revealed perifoveal telangiectasia mainly located in the superior and temporal area in the RE and in the inferior and temporal area in the LE with late intraretinal staining corresponding to the angiographic cystic macular edema (Figures 2(c), 2(d), 2(e), and 2(f)). FA examination confirmed the presence of telangiectasia in the anterior periphery and also the absence of vein abnormalities excluding a branch retinal vein occlusion.

SD-OCT (Spectralis Heidelberg Engineering, Germany) showed cystoid macular edema on both eyes with subretinal fluid in the RE and central macular thickness was measured to 538 *μ*m on RE and to 334 *μ*m LE (Figures 2(g) and 2(h)). Several microaneurysms within the inner retinal layers were also observed in this case. Autofluorescence images revealed in RE some hypoautofluorescent lesions in the temporal and inferior macular area, with a stellar distribution corresponding to the exudates. In the LE, the autofluorescence images revealed a large hypoautofluorescent lesion in the temporal area coexisting with smaller ones in the inferior area corresponding to photocoagulation scars (Figures 2(i) and 2(j)).

As in the case 1, we conclude the diagnosis of bilateral type 1 IMT since there was no medical history of diabetes or X-ray radiation and considering the absence of evidence of retinal vein occlusion on FA examination. Moreover the absence of typical intraretinal pseudocysts on OCT excluded the diagnosis of type 2 IMT. Despite a medical history of arterial hypertension, we did not consider the diagnosis of hypertensive maculopathy, since the absence of other retinal typical abnormalities and since the disease was under control. The patient was treated with anti-VEGF intravitreal injections in both eyes according to a PRN regimen without new focal laser photocoagulation since telangiectasia was located less than 1000 *μ*m to the fovea. One month after 3 anti-VEGF injections, visual acuity improved to 86 letters RE and 64 letters LE and OCT showed complete subretinal fluid resorption RE and partial macular edema resorption on both eyes. Central macular thickness reduced to 447 *μ*m RE and 295 *μ*m LE.

## 3. Discussion

Here we reported two cases with unusual presentation of bilateral type 1A IMT. Previous publications reported rare presentation of unilateral type 1 IMT associated with choroidal neovascularization [4, 5]. To our knowledge, the clinical presentation of our cases has never been previously described.

Before considering the diagnosis of type 1A IMT, we made sure to exclude other diagnoses. Indeed type 1 IMT must be differentiated from secondary telangiectasia caused by other retinal vascular diseases especially retinal venous occlusions, diabetic retinopathy, radiation retinopathy, Irvine-Gass syndrome, or hypertensive retinopathy. No medical history of diabetes or X-ray treatment was noted in our patients. In both cases FA showed no evidence of capillary dilatations related to a previous branch retinal vein occlusion and confirmed also the absence of late staining of the optic disc in case 1 excluding the diagnosis of Irvine-Gass syndrome. Moreover in case 2 we made sure that arterial hypertension was well-balanced.

According to the classification by Gass and Blodi [2], diagnosis of type 1 IMT is typically made in the presence of visible and exudative unilateral telangiectasia accompanied by microaneurysms. Our cases illustrated that a bilateral presentation does not exclude the diagnosis of type 1 IMT. Indeed, the frequency of the bilateral involvement is rare, around 6% of cases according to Gass and Blodi [2]. Moreover, among the 10 patients with aneurysmal telangiectasia in Yannuzzi's study, only one patient was found to have a bilateral disease [3].

Our cases may also suggest that middle-aged women can be affected with idiopathic type 1 Mac Tel, but less frequently. Indeed according to Gass and Blodi, type 1A IMT typically affects young males in 90% of cases with an average age of 39 years [2]. Moreover a recent study, including 8 patients with type 1A IMT, found a male predominance with 63% of cases and a mean age of 55.8 years [6].

Regarding the aspect of telangiectasia, it is not visible in fundus examination in case 1. However telangiectasia is typically visible in type 1 IMT and appears like aneurysmal lesions and occult presentation is usually observed in type 2 IMT [2, 3]. These observations may suggest that the absence of apparent vascular abnormalities in fundus examination does not exclude the diagnosis. In this case, FA may be contributive to diagnose infraclinical telangiectasia. Furthermore in case 1 we found any vascular abnormalities in the middle or anterior fundus periphery, as it is commonly observed in type 1 Mac Tel [1, 3]. In fact Gass and Blodi believed that type 1 IMT may be a localized form or part of the spectrum of congenital retinal telangiectasia or Coats' disease [2]. Similarly, Yannuzzi et al. consider aneurysmal telangiectasia to be a form of Coats' disease localized in the macula area [3].

We described two middle-aged women presenting with bilateral type 1A IMT and occult and without any vascular abnormalities in the middle or anterior fundus periphery in one case. Our cases emphasize that these unusual presentations do not exclude the diagnosis of type 1A IMT. Case 1 may probably be related to another pathophysiological mechanism than a localized Coats' disease.

The description of more cases of bilateral type 1A IMT would be useful in order to more precisely define their clinical presentation and their pathophysiological occurrence.
